# *Macaranga peltata* Alleviates Neuropsychiatric Disorders in Mouse Model and Computational Study

**DOI:** 10.1002/fsn3.71484

**Published:** 2026-01-23

**Authors:** Shaifullah Mansur Tanzil, Ahmed Azizul Hakim, Md Tasaffiul Islam, Farhan Tanvir, Israt Jahan, Arafat Faraque, Syed Mohammed Tareq, Md Areeful Haque, Md Amjad Hossen, Md. Shohel Al Faruk, Kazi Ashfak Ahmed Chowdhury, Mohammad Nazmul Islam

**Affiliations:** ^1^ Department of Pharmacy International Islamic University Chittagong Chittagong Bangladesh; ^2^ Department of Physiology, Biochemistry and Pharmacology Chattogram Veterinary and Animal Sciences University Chattogram Bangladesh; ^3^ Department of Pharmacy Jahangirnagar University Dhaka Bangladesh

**Keywords:** anxiety, depression, MEMPL, nociception, phytochemicals

## Abstract

This study investigated the neuropharmacological and analgesic effects of the methanol extract of *Macaranga peltata* leaves (MEMPL) in rodents, supported by computational modeling. Phytochemical and GC–MS analyses revealed alkaloids, flavonoids, phenols, glycosides, diterpenes, and other bioactive compounds. MEMPL (200 and 400 mg/kg) significantly increased open‐arm exploration in the elevated plus maze and reduced head dipping in the hole‐board test (*p* < 0.05), indicating anxiolytic activity. Dose‐dependent reductions in locomotor activity in the open‐field and hole‐cross tests (*p* < 0.05) suggested central nervous system depressant effects. Although MEMPL reduced immobility in both FST and TST, its effects were notably weaker than fluoxetine, indicating modest antidepressant‐like activity. MEMPL also demonstrated significant antinociceptive effects in acetic acid–induced writhing and formalin‐induced paw‐licking tests, likely via prostaglandin inhibition. Computational screening identified 2‐hydroxy‐6‐methylbenzaldehyde as a potential bioactive compound with strong binding to proteins involved in anxiety, depression, and nociception. Overall, these findings highlight MEMPL's broad neuropharmacological and analgesic potential, demanding further mechanistic and dose–response studies.

## Graphical abstract

The methanol extract of *Macaranga peltata* leaves (MEMPL) exhibited notable neuropharmacological and analgesic properties in rodents. Phytochemical and GC–MS analyses confirmed diverse bioactive compounds. MEMPL (200–400 mg/kg) showed anxiolytic, antidepressant, CNS depressant, and antinociceptive effects, comparable to standard drugs. Computational modeling identified 2‐hydroxy‐6‐methylbenzaldehyde as a potential active ligand targeting key neural receptors. These results suggest *M. peltata* as a promising source of phytochemicals for managing anxiety, depression, and pain‐related disorders.

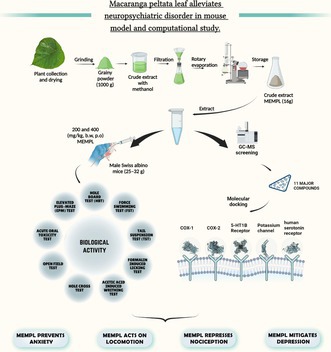

## 1 Introduction

Modern stressful lifestyles are associated with a wide range of pervasive psychological disorders, like anxiety and depression, which typically manifest in patients with low levels of monoamine neurotransmitters (Shao and Zhu 2020). According to recent statistics, nearly 1 in every 7 people worldwide—about 1.1 billion individuals—were living with a mental disorder. Among these, approximately 332 million suffered from depression and 359 million from anxiety (W.H.O. 2025). Clinically, serotonin–noradrenaline reuptake inhibitors (SNRIs), selective serotonin reuptake inhibitors (SSRIs), selective reversible inhibitors of monoamine oxidase A (RIMAs), and tricyclic antidepressants (TCAs) are suggested for the treatment of such disorders; meanwhile, the efficacy of such treatments may vary considering individual differences, as well as the prolonged use of these currently available synthetic medications, which have a propensity to cause major adverse effects, including impairments of the immunological, digestive, and respiratory systems, as well as a reduction in cognitive function, physical dependency, and tolerance (Liu et al. 2018). Several promising bioactive compounds derived from plant extracts serve as the foundation for pharmacological research and discovering novel molecules for the treatment of epilepsy, anxiety, and depression (Kaur et al. 2021; Emon et al. 2021; Mohammad et al. 2025; Shoaib et al. 2023; Küpeli Akkol et al. 2021). Notably, extracts from *Syzygium grande* , *Crataeva nurvala*, and *Blumea lacera* exhibited antidepressant, sedative, and anxiolytic‐like effects with suppression of locomotor activity in an animal model (Hossen et al. 2021; Moniruzzaman et al. 2018; Mamun et al. 2025). Considering this context, it is crucial to put scientific data on the pharmacological profile of therapeutic plants in order to ascertain their safety and efficacy, collaborating toward supporting their medicinal uses.

*Macaranga peltata* leaf is a resinous tree found in Bangladesh, Sri Lanka, India, northern Thailand, and other tropical countries, belonging to the Euphorbiaceae family. In Sri Lankan and Indian folk medicine, the leaves and bark are applied externally to reduce inflammation and treated internally for ailments associated with pain and oxidative stress‐related conditions. *M. peltata* was selected based on phytochemical evidence that the species and its close relatives ( *M. tanarius* , *M. indica* , *M. gigantea* ) contain prenylated flavonoids, phenolics, and stilbenoids with reported neuroprotective, anti‐inflammatory, and GABAergic activity. Specific compounds such as luteolin derivatives—identified previously in *M. peltata* —have demonstrated anxiolytic or antidepressant‐like effects in other plant models. These preliminary data provided a rational basis for investigating the anxiolytic, antidepressant, and analgesic potential of *M. peltata* (Honnappa et al. 2025; Magadula 2014). Hence, the current study was undertaken to objectively examine *Macaranga peltata*'s neuropharmacological potential in order to substantiate its traditional medicinal claims and investigate potential mechanisms of action on the central nervous system.

## 2 Materials and Methods

### 2.1 Chemicals and Drug

Methanol was purchased from Sigma Chemical Company in St. Louis, MO, USA. Standard drugs, that is fluoxetine HCl and diazepam, were bought from Square Pharmaceuticals Ltd., Bangladesh. Normal saline (0.9% NaCl) from Social Marketing Company Ltd., Bangladesh, and Tween 80 were obtained from BDH Chemicals (Leicestershire, UK). MEMPL was dissolved in saline with 1% Tween 80, whereas all the other drugs were dissolved in isotonic saline solution (NaCl 0.9%) prior to use. All other chemicals and reagents were bought from Merck (Darmstadt, Germany).

### 2.2 Plant Materials

*Macaranga peltata* leaves were collected from the rural area of Raozan, Chittagong, Bangladesh, in 2018. Dr. Sheikh Bokhtear Uddin, a prominent taxonomist and professor in the Department of Botany at the University of Chittagong, identified the sample, and a voucher specimen (MPL2018) was kept in the Department of Pharmacy at the International Islamic University. The International Plant Name Index (IPNI) was used to verify the plant's nomenclature.

### 2.3 Extract Preparation

Samples were cleaned and dried under shade at room temperature (23°C ± 0.5°C) and dried samples were ground to a coarse powder by a mechanical grinder. Thereafter, 1000 g of powder was successively extracted with methanol to yield crude extract. The filtrate was evaporated by rotary evaporator (RE200, Bibby Sterling, UK) under reduced pressure and temperature below 50°C. The yield value of the methanol extract (MEMPL) was noted as 16 g, and until the experiment was conducted, MEMPL was stored at 4°C.

### 2.4 Qualitative Phytochemical Assessment

The prevalence of several MEMPL secondary metabolites and phytochemicals was determined by using the following conventional methods (Dhanaraj and Vasanth Raj 2012).

### 2.5 Quantitative Phytochemical Assessment

#### 2.5.1 GC–MS Screening

The bioactive compounds in MEMPL were explored by using GC–MS with the electron impact ionization (EI) method on gas chromatography (GC‐17A, Shimadzu Corporation, Kyoto, Japan) and a mass spectrometer (GC–MS TQ 8040, Shimadzu Corporation, Kyoto, Japan). The DB‐1 (J&W) coating was applied to the fused capillary silica column (Rxi‐5 ms; 0.25 m film, 30 m long, and 0.32 mm internal diameter). The inlet temperature of the capillary was set at 250°C, and the oven temperature was set at 50°C for 1 min, 200°C for 2 min at 15°C/min, and 300°C for 7 min at 5°C/min. Using helium gas, the column flow rate was 1 mL/min at 53.5 kPa constant pressure. The temperature of the auxiliary (GC to MS interface) was set to 230°C for the ion source, 250°C for the interface, and 1.74 kV for the detector. With a scanning range of 50–600 m/z, the MS was tuned to Q3 scan mode. GC–MS analysis was performed on the prepared sample. The total GC–MS running time was 36.10 min, with a sample injection volume of 1 μL. The percentage of compound abundance was checked by applying the total ionic chromatogram (TIC), and all constituent peak regions were compared to the database in the GC–MS library version NIST 08‐S.

### 2.6 Animals

Male Swiss albino mice, about 25–32 g, aged 7–8 weeks, were purchased from Jahangirnagar University in Savar, Dhaka, Bangladesh. Mice were placed in polycarbonate cages, maintaining a standard laboratory environment (room temperature 23°C ± 2°C and humidity 55%–60%) in a 12‐h/daylight cycle. Mice were provided easy accessibility to a livestock pellet meal as well as water from the tap. Prior to beginning the trial, mice spent 2 weeks adapting to the laboratory setting (Oecd 2002).

### 2.7 Acute Oral Toxicity Test

MEMPL was assessed for acute toxicity using the Organization for Economic Cooperation and Development's guidelines (OECD: Guidelines 423; Fixed‐Dose Method). Five groups of five mice were given oral doses ranging from 100, 200, 400, 1000, 2000, and 4000 mg/kg BW following an overnight fast. Mice were fasted for the whole night before the extract was given, and their meals were postponed for 3 to 4 h. Meals were not given for an additional 1–2 h following treatment, and individual mice were monitored for the first half hour following dosage and then at regular intervals for a period of 24 h (with special attention for the first 4 h), with a focus on behavioral alteration, allergic syndromes (skin rash, itching, swelling), and mortality over the next 72 h and until completion by 14 days (Oecd 2002). The effective therapeutic dose was found to be one‐tenth of the median lethal dose (LD50 > 4.0 g/kg) (Ahmed et al. 2021).

### 2.8 Experimental Design

Mice were randomly composed into six groups. Each group has six mice, and all mice were intervened by the following methods: Group A (NC) served as the normal control receiving vehicle (1% Tween 80 in DW, 10 mL/kg, p.o.); Groups B and C received MEMPL 200 and 400 (mg/kg, b.w., p.o.), respectively; and Groups D, E, and F served as positive controls, receiving standard drugs diazepam (1 mg/kg, b.w., i.p.) (DM‐1) used for anxiolytic assay, fluoxetine (20 mg/kg, b.w., i.p.) (FN‐20) used for antidepressant assay, and diclofenac Na (10 mg/kg, p.o.) (DS‐10) used for analgesic assay, respectively.

### 2.9 Anxiolytic Assessment

#### 2.9.1 Elevated Plus‐Maze (EPM) Test

The modified version of Lister's verified mouse experiment is called the Elevated plus‐maze test (Uddin et al. 2018). The EPM is made up of two open arms without walls (35 × 5 cm^2^) and 2 enclosed arms by walls (30 × 5 × 15 cm^3^) that are both deployed from an identical floor (5 × 5 cm^2^). Black paint has been applied to the wood interior surfaces of the closed arms. The entire maze is raised 50 cm above the basement. For the purpose of promoting exploration, an edge (0.25 cm) was added to the open arm's perimeter. Mice were treated with samples according to the design of the experiment, and each of them was put into the maze's center facing one of the enclosed arms after a 30‐min treatment with the NC, DM‐1, MEMPL 200, and MEMPL 400. The number of entries and the total time spent in the open arm were recorded throughout the entire duration of a 5‐min period. The experiment had been carried out in a calm environment, assuring precise results.

#### 2.9.2 Hole Board Test (HBT)

The hole‐board test (HBT) is a frequently employed method for assessing the anxiolytic impact on a rodent model (Takeda et al. 1998). The test apparatus in this model was a flat platform with an enclosed area (20 × 40 cm^2^), which was placed 15 cm over the base in a pattern of grids with 16 holes (diameter 3 cm). The treatment was given according to the experimental design, and after half an hour, the mice were placed in the center of the platform and allowed to walk around freely. Mice were then monitored for 5 min to see how long it took them to lower their heads through the hole.

### 2.10 Locomotion Determination

#### 2.10.1 Open Field Test (OFT)

The open field test, which was previously explained (Seibenhener and Wooten 2015) was used to evaluate MEMPL's spontaneous locomotor activities. In order to give the mice time to become habituated to the experiment room, they were brought in at least an hour preceding every test. The OFT device consisted of a square box (50 × 50 × 40 cm) with its base split by two colored (black and white) square blocks (10 × 10 cm). After administering the treatments to the different groups, as detailed in the part that focuses on the testing methodology, mice were then put in the device, and its motions for 3 min were recorded at 0, 30, 60, 90, and 120 min into the entire study. Each one had been placed individually inside the center of the apparatus, and the number of squares it crossed with all four of its paws during that period was tracked. Between each trial, the OFT area had been meticulously washed in order to ensure that mice wouldn't be affected by the prior session's feces and pee scent.

#### 2.10.2 Hole Cross Test (HCT)

In earlier research, Takagi et al. conducted an investigation of the locomotor and exploratory behaviors using equipment known as hole‐cross test equipment (Takagi et al. 1971). A cardboard container measuring 30 by 20 by 14 cm with a 3‐cm hole in the middle was set at a height of 7.5 cm. The group was treated as indicated in the part that focuses on the experimental methodology. Each mouse was treated, then put in the device on its own, where the total number of holes crossed was recorded every 3 min for the first 30, 60, 90, and 120 min.

### 2.11 Antidepressant Evaluation

#### 2.11.1 Force Swimming Test (FST)

The most prevalent method to gauge the potency of antidepressants is the forced swimming test (FST) (Yankelevitch‐Yahav et al. 2015). Mice were kept at 25°C ± 1°C for 30 min, followed by a 6‐min period of observation in a translucent glass container (55 cm height, 30 cm diameter) that contained 19 cm of freshwater. The last 4 min of each animal's six‐minute observation time were noted as immobility and latency phases, while the first 2 min were set aside for acclimating to the equipment. According to the experimental design, mice were treated, and each animal was only required to keep its head above water to qualify as immobile for the duration of the trial.

#### 2.11.2 Tail Suspension Test (TST)

The duration of time spent immobile was assessed using the TST as described to gauge depressive‐like behavior (Steru et al. 1985). The experimental plan involved randomly administering different treatments to animals. Mice were held up 50 cm above the floor in a plywood suspension box with sticky tape after receiving treatments for 60 min. The tape was placed approximately 1 cm from the point of the tail. Each mouse's total immobility time was measured for the final 4 min within the 6‐min experiment.

### 2.12 Antinociceptive Activities

#### 2.12.1 Formalin Induced Licking Test

The formalin‐induced biphasic method was widely utilized in the rodent model to decipher the anti‐nociception effect (Shibata et al. 1989). A 20 μL dosage of 2.5% formalin solution, prepared from 0.9% saline solution, was injected into the sub‐plantar region of the right hind paw of mice. The pain was assessed by how frequently the mice licked and bit their paws after being injected. After formalin injection, the first phase of response is defined as data collected for the first 5 min, and the second phase as data collected for the subsequent 15–30 min. The initial and final stages of the pain response correspond to inflammatory and neurogenic pain responses, respectively. The following calculation was carried out to unravel the % of inhibition in licking time for anti‐nociceptive activity:

Percentage of inhibition = [(T−C)/C] × 100, where C is the mean number of licks (control) and T is the mean number of licks (treatment).

#### 2.12.2 Acetic Acid‐Induced Writhing Test

Different groups of mice were treated according to the experimental design. After 30 min of treatment, the mice were given an intraperitoneal injection of 1% (v/v) acetic acid (10 mL/kg body weight). Abdominal constraints were noticed for 10 min following an acetic acid injection at 5 min, and the results were compared to the control group (Koster 1959). The given equation was used to figure out the % of writhing suppression for the anti‐nociceptive action. The percentage of writhing inhibition is calculated as [(T−C)/C] × 100, where C = the average number of writhing (control) and T = the average number of writhing (treatment).

### 2.13 Computational Studies

#### 2.13.1 Pharmacokinetic and Toxicity Studies

QikProp (Schrödinger Release 2017–1: QikProp, Schrödinger LLC, New York, NY, USA) was used to unveil the pharmacokinetic properties of bioactive compounds from MEMPL. Lipinski's rule of fives (Lipinski et al. 2001) and Veber's rule of three (Veber et al. 2002) were used to assess the absorption, distribution, metabolism, excretion, and toxicity (ADME/T) properties of MEMPL's key bioactive molecules. On top of that, the QikProp application revealed a number of pharmacological aspects to explore drug‐like abilities.

#### 2.13.2 Molecular Docking Analysis

To unveil anti‐nociceptive, anxiolytic, and antidepressant activities, the following major proteins, COX‐1 (PDB ID: 2YOE), COX‐2 (PDB ID: 3HS5), potassium channel (PDB ID: 4UUJ), 5‐HT1B (PDB ID: 4IAQ), and human serotonin receptor (PDB ID: 5I6X) were chosen according to literature study, and their crystal structures were downloaded from the RCSB Protein Data Bank (PDB), a digital repository (https://www.rcsb.org/). Finally, the online program PockDrug (Hussein et al. 2015) was used to select the most effective binding locations. Furthermore, depending on abundance, the chemical structure of the essential compounds found in MEMPL by GC–MS was extracted from the PubChem library (https://pubchem.ncbi.nlm.nih.gov/) based on abundance.

### 2.14 Ethical Considerations

The Institutional Animal Ethics Committee of the Department of Pharmacy, International Islamic University Chittagong, approved ethical animal handling protocols (IIUC/PHARM‐AEC‐52/10–19).

### 2.15 Statistical Analysis

SPSS 22.0 (IBM, New York, USA) and GraphPad Prism Data Editor for Windows, Version 8.4.3, were used to figure out the significant differences and make graphs. Treatments were significantly compared with normal control by using one‐way analysis of variance (ANOVA) followed by Dunnett's test. Each of the values is represented as mean ± standard error of mean (SEM). Statistical significance was determined with *p*‐values (cp < 0.05, bp < 0.01, and ap < 0.001).

## 3 Results

### 3.1 Qualitative Phytochemical Screening

The current research has identified a number of secondary metabolites in MEMPL (Table 1), including diterpenes, alkaloids, proteins, amino acids, carbohydrates, glycosides, flavonoids, and phenols.

**TABLE 1:** Preliminary phytochemical status of MEMPL.

Classes of secondary metabolites	Standards	Observation
Alkaloids	Piperine	++
Carbohydrates	Glucose	+
Glycosides	Fructoside	+
Flavonoids	Quercetin	+
Tannins	Tannic Acid	—
Saponins	Vina‐ginsenosides‐R5	—
Phenols	Catechol	+
Protein and amino acids	BSA	++
Diterpenes	Retinol	++

*Note:* Bioavailability key: indicates no presence, + indicates low concentration, and ++ indicates fairly high concentration.

### 3.2 GC–MS Analysis

**FIGURE 1:**
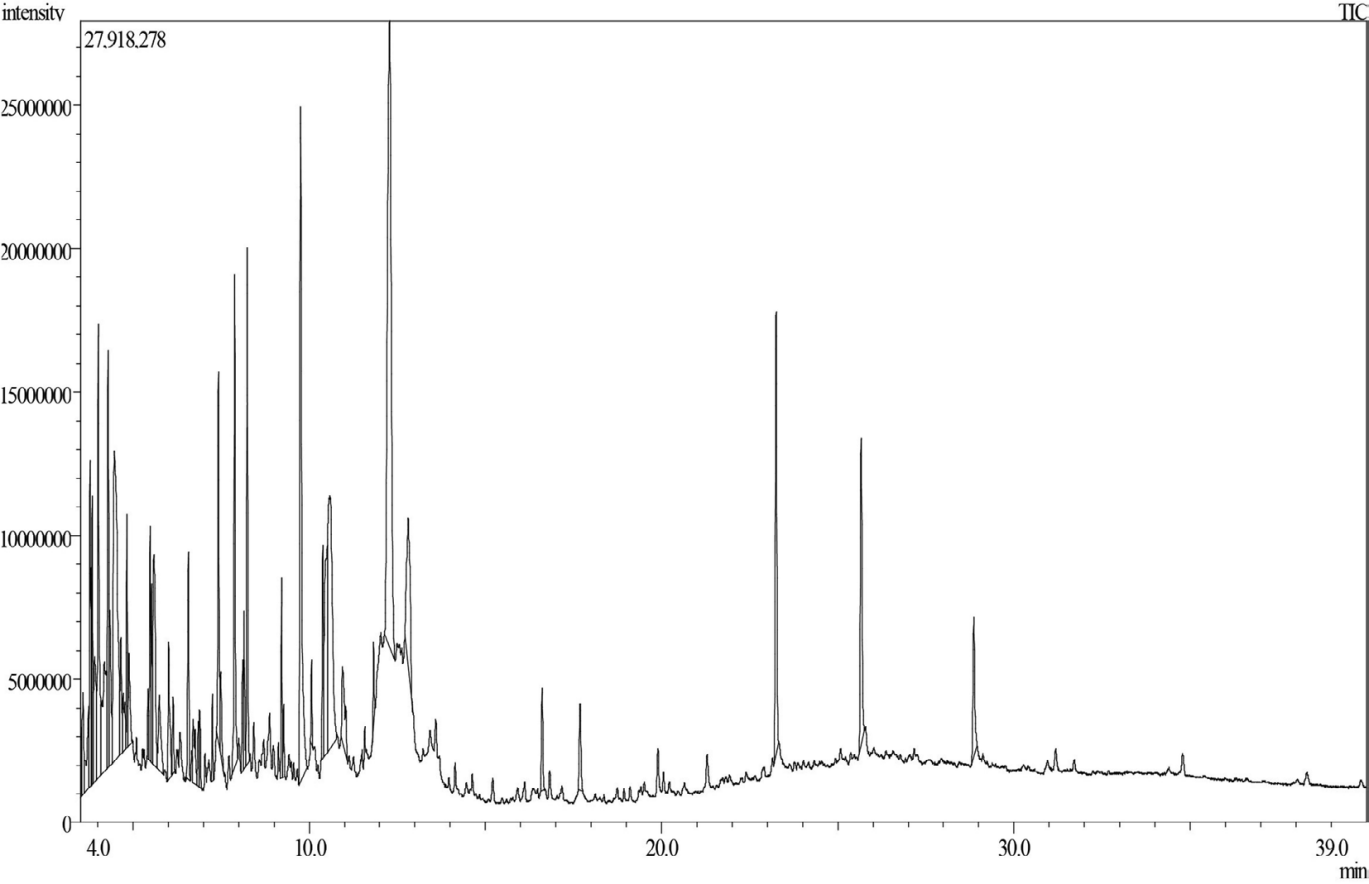
Total ionic chromatogram (TIC) of bioactive compounds of MEMPL by GC–MS analysis.

The GC–MS screening of MEMPL found approximately 30 bio‐metabolites with retention times ranging from 3.78 to 28.86, as shown in Table 2, with the chromatogram in Figure 1. The essential bio‐metabolites observed in the MEMPL include 1,2,3,5‐Cyclohexanetetrol, (1.alpha.,2.beta.,3.alpha.,5.beta.)‐ (13.26%), 9‐Octadecenamide, (Z)‐ (3.76%), Ethanamine, 2‐methoxy‐N‐(2‐methoxyethyl)‐N‐methyl‐ (3.17%), 4H‐Pyran‐4‐one, 2,3‐dihydro‐3,5‐dihydroxy‐6‐methyl‐ (2.42%), Thymine (1.48%), Benzaldehyde, 2‐hydroxy‐6‐methyl‐ (1.47%), beta.‐D‐Glucopyranose, 1,6‐anhydro‐ (1.26%), 2,4‐Dihydroxy‐2,5‐dimethyl‐3 (2H)‐furan‐3‐one (1.35%).

**TABLE 2:** Secondary bioactive compound found in the MEMPL by GC–MS screening.

Sl.	RT (min)	Compounds	MF	MW (g/mol)	Area (%)	Chemical class
1.	3.78	3,4‐Furandiol, tetrahydro‐, cis—	C_4_H_8_O_3_	104.10	1.74	Alcohol
2.	3.909	Fluoroacetic acid	C_2_H_3_FO_2_	78.04	1.66	Halogenated carboxylic acid
3.	4.017	2‐Furanmethanol	C_5_H_6_O_2_	98.10	3.65	Furfural alcohol
4.	4.292	Ethanamine, 2‐methoxy‐N‐(2‐methoxyethyl)‐N‐methyl—	C_4_H_11_NO	89.14	3.17	Monoalkylamine
5.	4.474	(S)‐(+)‐2‐Amino‐3‐methyl‐1‐butanol	C_5_H_13_NO	103.16	7.35	Alkanolamine
6.	5.486	2,4‐Dihydroxy‐2,5‐dimethyl‐3 (2H)‐furan‐3‐one	C_6_H_8_O_4_	144.12	1.35	Furan
7.	6.571	Thymine	C_5_H_6_N_2_O_2_	126.11	1.48	Hydroxypyrimidine
8.	7.424	4H‐Pyran‐4‐one, 2,3‐dihydro‐3,5‐dihydroxy‐6‐methyl—	C_6_H_8_O_4_	144.12	2.42	Triterpene
9.	7.882	Catechol	C_6_H_6_O_2_	110.11	3.48	Phenol
10.	8.24	5‐Hydroxymethylfurfural	C_6_H_6_O_3_	126.11	2.97	Aldehyde
11.	9.216	2‐Methoxy‐4‐vinylphenol	C_9_H_10_O_2_	150.17	1.06	Phenol
12.	9.754	1,2,3‐Benzenetriol	C_7_H_10_O_6_S	222.22	6.7	Phenol
13.	10.385	2‐hydroxy‐6‐methylbenzaldehyde	C_8_H_8_O_2_	136.15	1.47	Benzaldehyde
14.	10.948	beta.‐D‐Glucopyranose,1,6‐anhydro—	C_6_H_10_O_5_	162.14	1.26	Monosaccharide
15.	12.279	1, 2, 3, 5‐Cyclohexanetetrol, (1.alpha., 2.beta., 3.alpha., 5.beta.)—	C_6_H_12_O_4_	148.16	13.26	Cyclohexane
16.	23.248	9‐Octadecenamide, (Z)—	C_18_H_35_NO	281.5	3.76	Monounsaturated fatty acid

Abbreviations: MF, molecular formula; MW, molecular weight; RT, retention time.

### 3.3 MEMPL Prevents Anxiety

**FIGURE 2:**
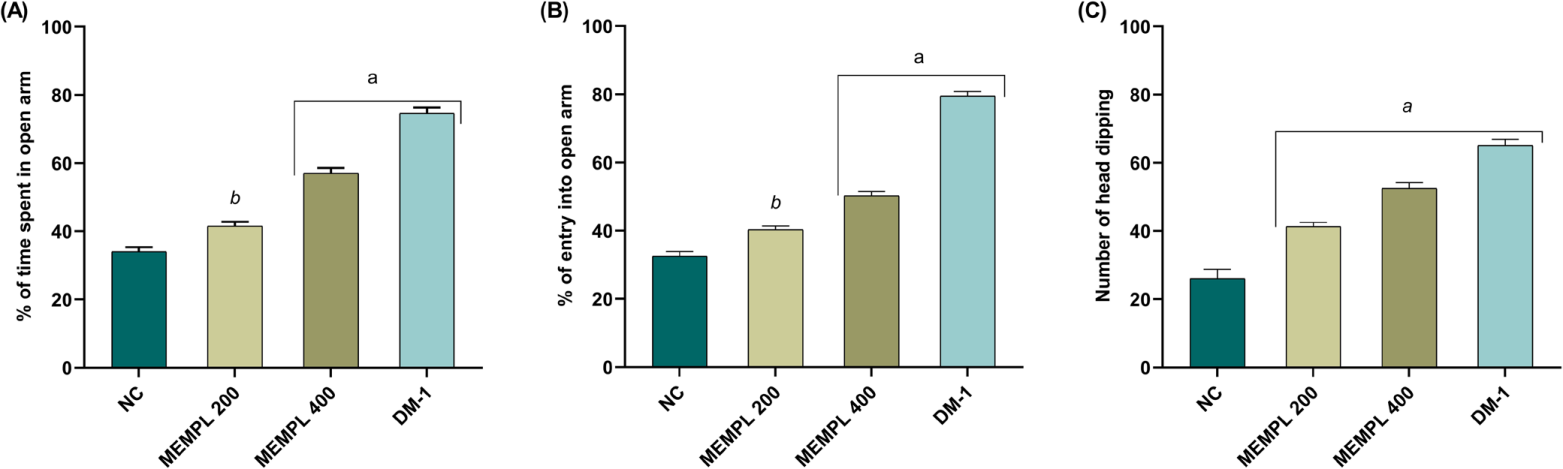
MEMPL alleviates anxiety in mice (A) % of a period of time spent in the open arm in the EPM, (B) % of entry in the open arm in the EPM, and (C) number of head dips in the HBT on mice. Values are shown as mean ± SEM, where *n* = 6 mice/group, ^c^ *p* < 0.05, ^b^ *p* < 0.01 and ^a^ *p* < 0.001 indicate significant differences from control. EPM = Elevated Plus Maze; HBT = Hole Board Test; NC = Normal Control; MEMPL = Methanol Extract of Macaranga Peltata Leaves; DM‐1 = Diazepam 1 mg/kg.

MEMPL demonstrated significant anxiolytic‐like effects in both the Elevated Plus Maze (EPM) and Hole Board Test (HBT) models. Treatment with MEMPL (200 and 400 mg/kg) and diazepam (1 mg/kg) significantly (^a^ *p* < 0.001 and ^b^ *p* < 0.01) increased the time spent in the open arms (41.50 ± 1.33, 57.00 ± 2.00, and 74.66 ± 1.66, respectively; Figure 2A). Similarly, the percentage of entries into the open arms (40.33 ± 1.02, 50.33 ± 1.20, and 79.50 ± 1.38, respectively) was markedly elevated (^a^ *p* < 0.001 and ^b^ *p* < 0.01; Figure 2B). In the HBT (Figure 2C), MEMPL (200 and 400 mg/kg) and diazepam (1 mg/kg) significantly (^a^ *p* < 0.001) increased the number of head dips (41.33 ± 1.22, 52.50 ± 1.72, and 65.16 ± 1.72, respectively), further supporting anxiolytic‐like activity. Overall, these results indicate that MEMPL mitigates anxiety‐like behavior, as evidenced by increased open‐arm exploration and head‐dipping frequency. However, since MEMPL concurrently reduced locomotor activity in subsequent tests, these anxiolytic‐like outcomes may partially reflect CNS depressant action rather than a purely anxiolytic mechanism. The magnitude of response observed with MEMPL was notably lower than that of diazepam, indicating limited efficacy relative to the standard drug.

### 3.4 MEMPL Acts on Locomotion

**FIGURE 3:**
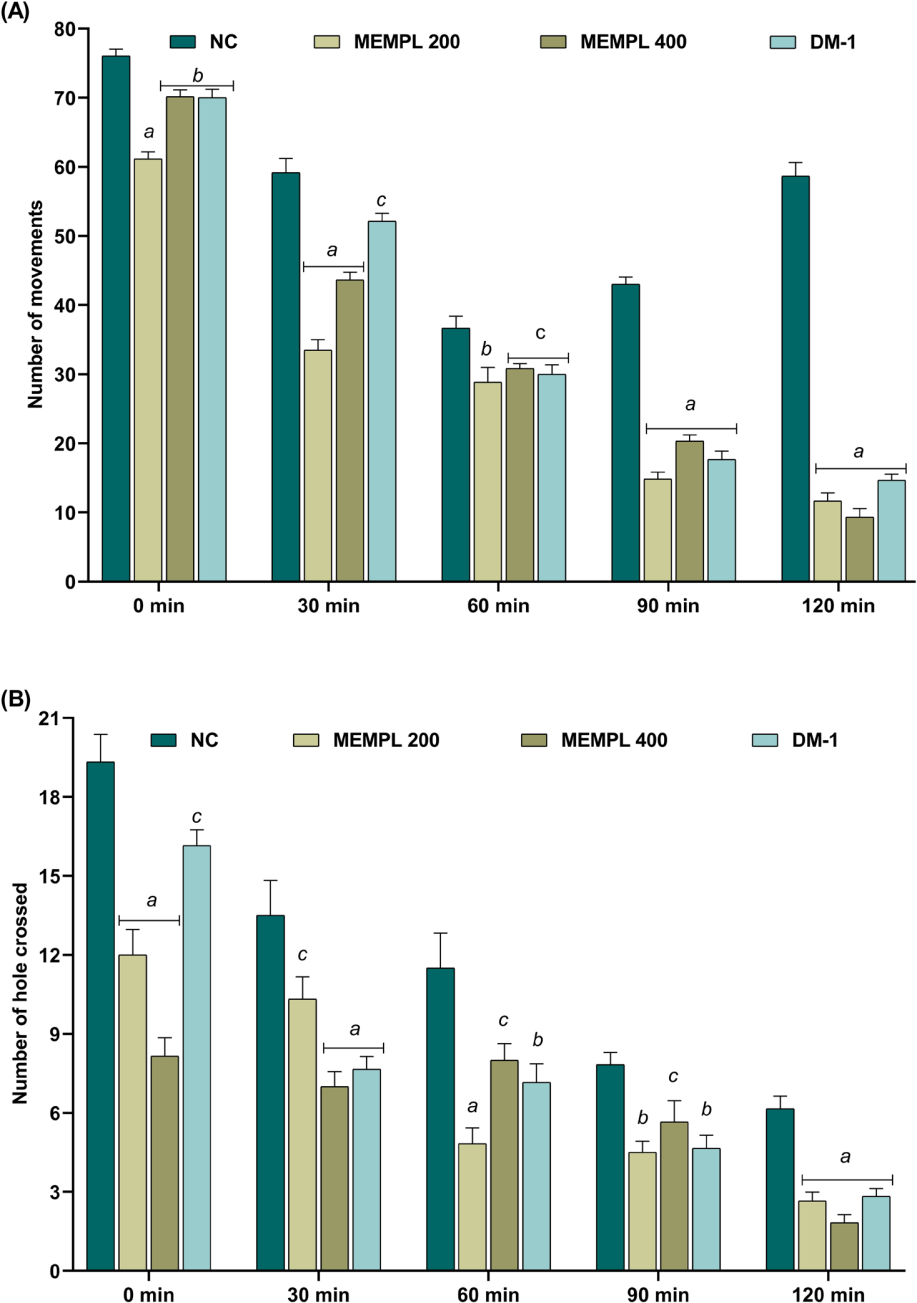
(A) MEMPL attenuates locomotor action in the OFT on mice (B) of MEMPL modulates locomotor action in the HCT on mice. Data are presented as mean ± SEM, where *n* = 6 mice/group, ^c^ *p* < 0.05, ^b^ *p* < 0.01 and ^a^ *p* < 0.001, significantly different from control. OFT = Open Field Test; HCT = Hole Cross Test; MEMPL = Methanol Extract of *Macaranga peltata* Leaves; DM‐1 = Diazepam 1 mg/kg.

MEMPL modulated locomotor activity in the central nervous system (CNS), as assessed by the Open Field Test (OFT) and Hole Cross Test (HCT). In the OFT (Figure 3A), MEMPL caused a significant, dose‐dependent decline in movement (^c^ *p* < 0.05). Reduced locomotion was evident from the second observation phase and persisted up to 120 min. Mice treated with MEMPL (200 and 400 mg/kg) and diazepam (1 mg/kg) showed significantly reduced movement counts (33.5 ± 1.52, 43.66 ± 1.11, and 52.16 ± 1.10, respectively) compared with control. Similarly, in the HCT (Figure 3B), MEMPL significantly reduced locomotor activity. The number of hole crossings at 30 min (10.33 ± 0.84, 7.00 ± 0.57, and 7.66 ± 0.49 for MEMPL 200 mg/kg, MEMPL 400 mg/kg, and diazepam, respectively) and at 120 min (2.66 ± 0.33, 1.83 ± 0.30, and 2.83 ± 0.30, respectively) was lower than control (6.16 ± 0.47). These findings indicate sedative or CNS depressant properties of MEMPL, which should be considered when interpreting its behavioral effects in the anxiety and depression models.

### 3.5 MEMPL Mitigates Depression

**FIGURE 4:**
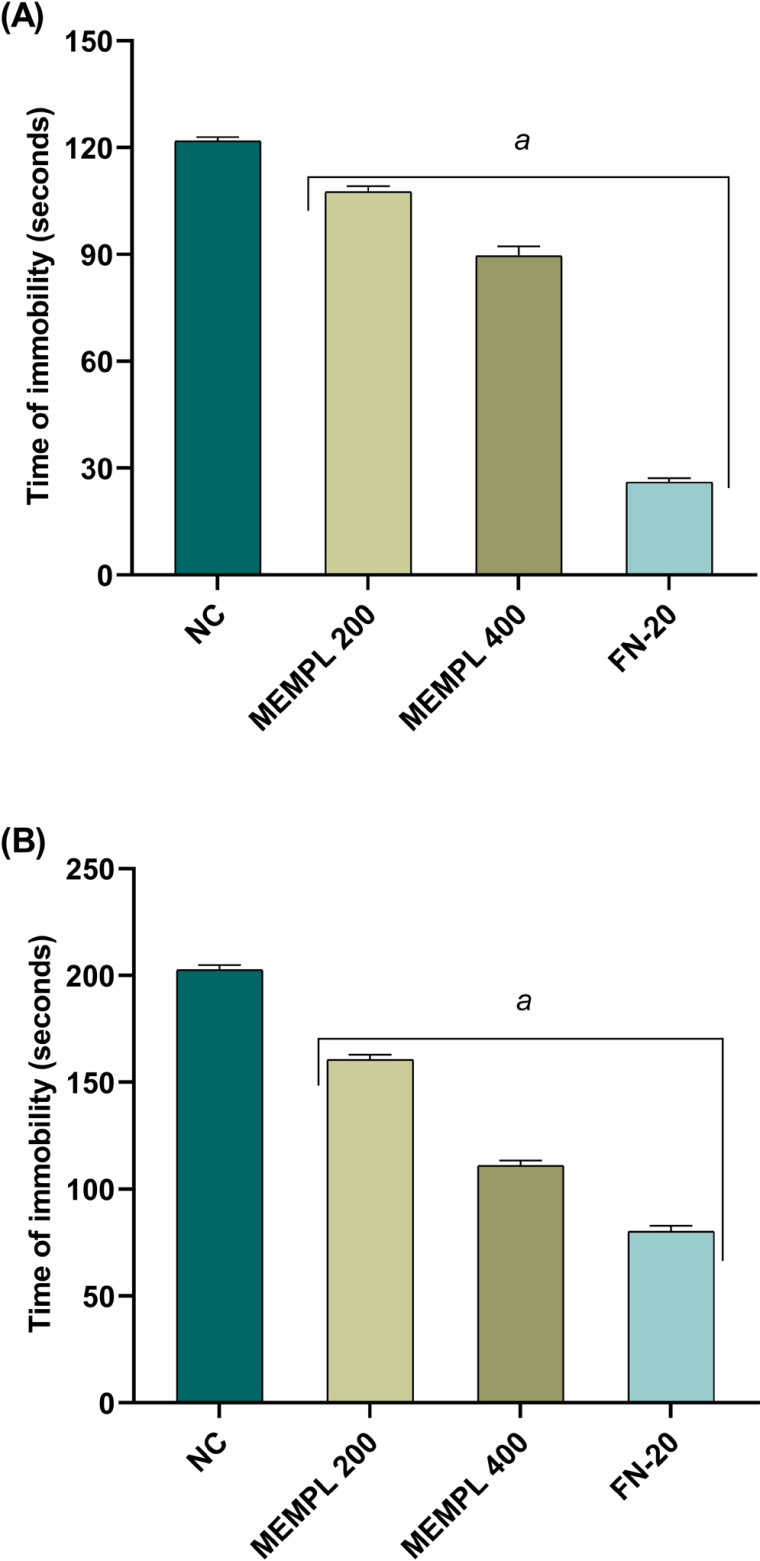
MEMPL modulates depression in (A) FST and (B) TST on mice. Values are displayed as mean ± SEM, where *n* = 6 mice/group, ^c^ *p* < 0.05, ^b^ *p* < 0.01 and ^a^ *p* < 0.001, suggesting significant difference from the control. FST = Forced Swimming Test; TST = Tail Suspension Test; MEMPL = Methanol Extract of *Macaranga peltata* Leaves; FN‐20 = Fluoxetin HCl 20 mg/kg.

The antidepressant activity of MEMPL was assessed using the Forced Swim Test (FST) and Tail Suspension Test (TST). In the FST (Figure 4A), treatment with MEMPL (200 and 400 mg/kg) and fluoxetine (20 mg/kg) significantly (^a^ *p* < 0.001) reduced immobility time (107.5 ± 1.6, 89.50 ± 2.8, and 26.00 ± 1.0, respectively) compared with control (121.83 ± 1.16). In the TST (Figure 4B), MEMPL and fluoxetine similarly decreased immobility (160.5 ± 2.7, 111.0 ± 2.0, and 80.16 ± 2.72, respectively; ^a^ *p* < 0.001) relative to control (202.5 ± 2.2). Although the extract demonstrated significant behavioral improvement, its efficacy was markedly lower than fluoxetine. The antidepressant‐like and anxiolytic responses may be associated with the presence of phytochemicals such as flavonoids, tannins, and phenolics, which have been reported to modulate neurotransmission. However, since no fractionation or receptor‐binding assays were performed, this relationship remains speculative and requires further mechanistic validation.

### 3.6 MEMPL Represses Nociception

**FIGURE 5:**
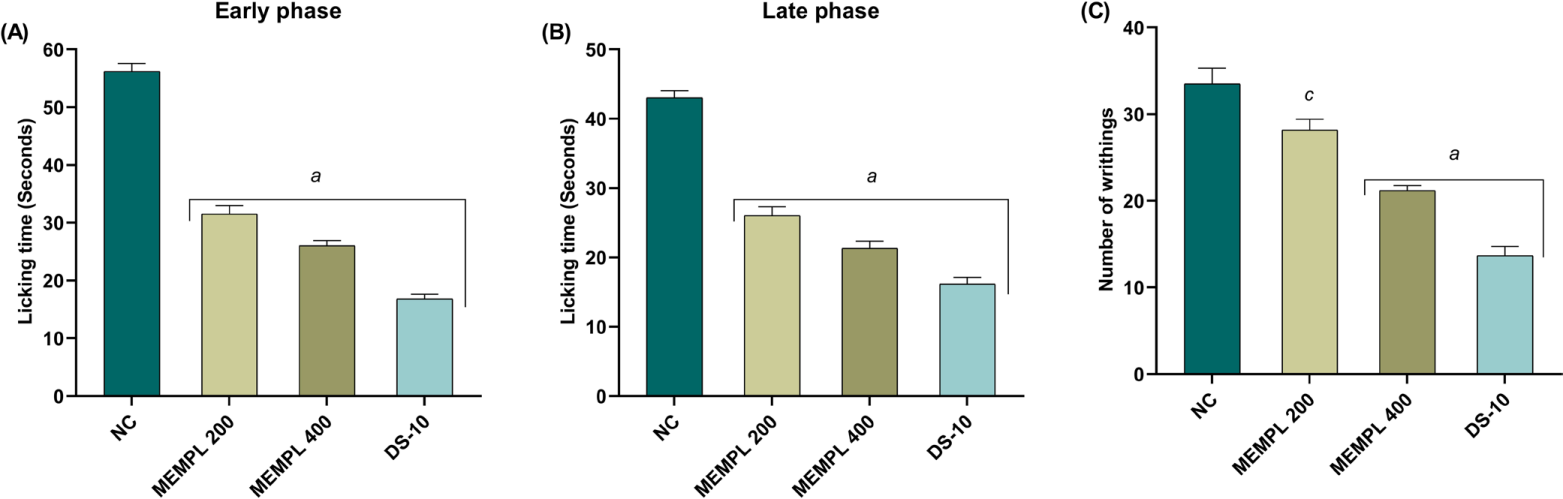
MEMPL suppresses formalin‐induced nociception (A) early phase and (B) late phase (C) acetic acid‐induced nociception. The values are provided as mean ± SEM, where *n* = 6 mice/group, and ^c^ *p* < 0.05, ^b^ *p* < 0.01 and ^a^ *p* < 0.001 indicating significant difference from the control. MEMPL = methanol extract of *Macaranga peltata*; DS‐10 = diclofenac Na 10 mg/kg.

The effect of MEMPL on pain perception was evaluated using the formalin‐induced paw licking test and the acetic‐induced writhing test. Treatment with MEMPL (200 and 400 mg/kg) and diclofenac sodium (10 mg/kg) effectively (^a^ *p* < 0.001) lowered the paw licking time (31.5 ± 1.5, 26 ± 1, and 16.83 ± 0.79, respectively) in the neurogenic phase, compared to the control group (56.16 ± 1.40) in the formalin‐induced paw licking test (Figure 5A). Furthermore, MEMPL treatment (200 and 400 mg/kg) and the reference drug diclofenac sodium (10 mg/kg) were found to be more effective (^a^ *p* < 0.001) in reducing paw‐licking time (26 ± 1.31, 21.33 ± 0.98, and 16.16 ± 0.94, respectively) in the inflammatory phase, compared to the control group (43 ± 1.06) (Figure 5B). At the same time, the results also show (Figure 5C) that high doses of MEMPL and the reference drug markedly (^a^ *p* < 0.001) lessened the number (21.16 ± 0.60 and 13.66 ± 1.05) of writhing, but low doses were comparatively (28.16 ± 1.24) less significant (^c^ *p* < 0.05) in the acetic‐induced writhing test, while these treatments compared to the control group (33.50 ± 1.82). Overall, these results suggest that MEMPL has promising anti‐nociceptive activity with the potential to reduce pain sensation.

### 3.7 Major Bioactive Compounds Selected by Pharmacokinetic Properties

The QikProp ADME/T prediction tool was used to evaluate the compounds' potential to serve as biologically active therapeutics in vivo. The research found that 11 of 30 compounds follow Lipinski's and Veber's rules, which are frequently used criteria to assess a compound's drug‐likeness (Table 3). Furthermore, the study also unfolded several physiochemical properties (Table 4) of the compounds, including their aqueous solubility, blood–brain partition coefficient, Caco‐2 cell permeability, K+ channel blockage, MDCK cell permeability, octanol/water partition coefficient, serum protein binding, and skin permeability. Based on these findings, the compounds might be intriguing therapeutic candidates, although more detailed research is needed.

**TABLE 3:** Pharmacokinetic characteristics of the identified bioactive compounds in MEMPL.

Compounds	Lipinski rules				Lipinski's violation (≤ 1)	Veber rules	
	MW (< 500)	HBA (< 10)	HBD (< 5)	Log *p* (≤ 5)		Nrb (≤ 10)	TPSA (≤ 100 Å)
3,4‐Furandiol, tetrahydro‐, cis—	104.1	3	2	−1.4	0	0	49.7
Propanenitrile,3‐(methylthio)—	101.17	2	0	0.6	0	2	49.1
Fluoroacetic acid	78.04	3	1	0.2	0	1	37.3
2‐Furanmethanol	126.15	2	1	1.1	0	2	33.4
2,4‐Dihydroxy‐2,5‐dimethyl‐3 (2H)‐furan‐3‐one	144.12	4	2	−0.1	0	0	66.8
Thymine	126.11	2	2	−0.6	0	0	58.2
4H‐Pyran‐4‐one, 2,3‐dihydro‐3,5‐dihydroxy‐6‐methyl	144.12	4	2	−0.4	0	0	66.8
5‐Hydroxymethylfurfural	126.11	3	1	−0.6	0	2	50.4
2‐Methoxy‐4‐vinylphenol	150.17	2	1	2.4	0	2	29.5
Benzaldehyde, 2‐hydroxy‐6‐methyl—	136.15	2	1	1.9	0	1	37.3
1, 2, 3, 5‐Cyclohexanetetrol, (1.alpha., 2.beta., 3.alpha., 5.beta.)—	148.16	4	4	−1.8	0	0	80.9

Abbreviations: HBA, hydrogen bond acceptor; HBD, hydrogen bond donor; Log P, liphophilicity; MW, molecular weight; nRB, number of rotatable bonds; TPSA, topological polar surface area.

**TABLE 4:** Demonstration of drug‐like features of the key bio‐metabolites using QikProp.

Compounds	QPlogPo/w (−2–6.5)	QPlogBB (−3–1.2)	QPlogS (−6.5–0.5)	QPlogKhs (−1.5–1.5)	QPlogHERG (concern < −5)	QPPCaco‐2 (< 25 poor, > 500 great)	QPPMDCK (< 25 poor > 500 great)	QPlogKp (−8 to −1)	Predicted CNS effect (−2) (inactive to (+2) active)	% of human oral absorption (< 25% is poor and > 80% is high)
3,4‐Furandiol, tetrahydro‐, cis—	−0.748	0.028	0.21	−0.812	−1.132	1573.242	807.351	−3.073	−1	66.823
Propanenitrile, 3‐(methylthio)—	0.028	0.139	−0.507	−0.875	−3.045	2602.102	2019.198	−2.648	0	88.237
Fluoroacetic acid	−0.563	−0.431	−0.827	−1.007	−4.722	1134.149	912.572	−3.253	0	78.322
2‐Furanmethanol	0.445	0.313	−0.744	−0.729	−2.73	4903.288	2758.577	−2.113	1	95.605
2,4‐Dihydroxy‐2,5‐dimethyl‐3 (2H)‐furan‐3‐one	−0.333	−0.032	−0.052	−0.939	−2.437	2074.855	1088.871	−2.743	0	84.363
Thymine	0.082	−0.689	−1.643	−0.684	−4.649	683.659	327.968	−3.776	−1	78.167
4H‐Pyran‐4‐one, 2,3‐dihydro‐3,5‐dihydroxy‐6‐methyl	−0.254	−0.603	−1.277	−0.869	−4.566	905.191	444.213	−3.443	0	78.38
5‐Hydroxymethylfurfural	−1.201	−0.425	0.299	−1.058	−1.937	551.3	259.909	−3.862	−1	68.979
2‐Methoxy‐4‐vinylphenol	1.121	0.165	−0.87	−0.497	−2.338	3640.412	1999.343	−2.268	1	100
2‐hydroxy‐6‐methylbenzaldehyde	0.813	0.041	−0.404	−0.584	−1.924	2341.295	1240.762	−2.641	1	92.013
1, 2, 3, 5‐Cyclohexanetetrol, (1.alpha., 2.beta., 3.alpha., 5.beta.)	−1.263	−0.531	−0.395	−0.771	−1.967	332.636	150.541	−4.384	−1	51.733

Abbreviations: QOlogBB, predicted blood–brain partition coefficient; QPlogHERG, K+ channel blockage; QPlogKhsa, serum protein binding; QPlogKp, skin permeability in cm/h; QPlogPo/w, predicted octanol/water partition coefficient; QplogS, predicted aqueous solubility, S in mol/dm −3; QPPCaco‐2, apparent Caco‐2 (a gut blood barrier model) cell permeability (nm/s); QPPMDCK, apparent MDCK (a blood–brain barrier model) cell permeability (nm/s).

### 3.8 Bioactive Compounds of MEMPL Act on Key Receptors

**FIGURE 6:**
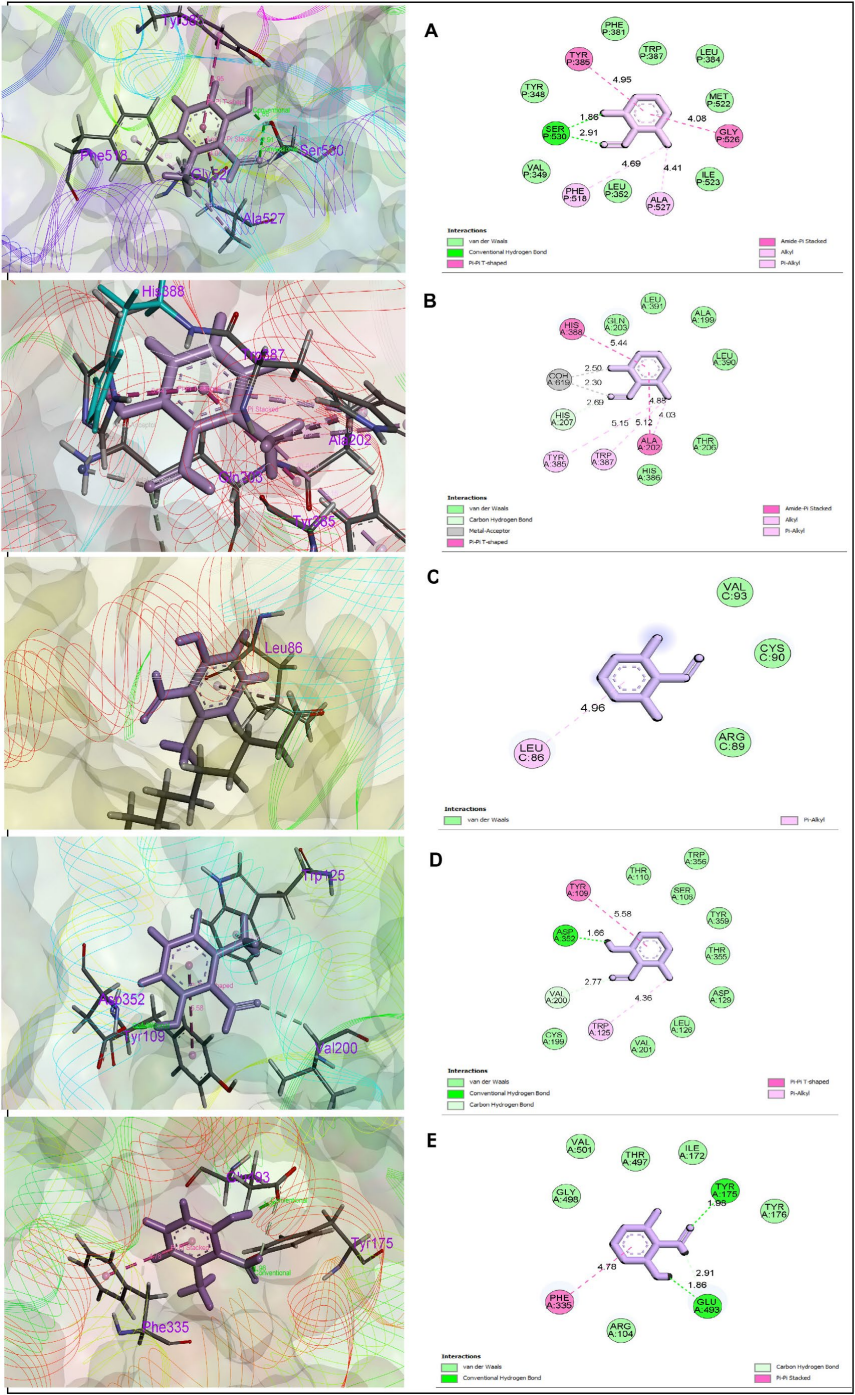
Binding modes and interaction profiles of 2‐hydroxy‐6‐methylbenzaldehyde with the selected receptors. The figure presents the 3D binding poses (left panels) and 2D interaction diagrams (right panels) of 2‐hydroxy‐6‐methylbenzaldehyde docked into five receptor targets: (A) 2OYE, (B) 3HS5, (C) 4UUJ, (D) 4IAQ, and (E) 5I6X.

Major 11 compounds interacted with three major proteins including COX‐1 (PDB ID: 2YOE), COX‐2 (PDB ID: 3HS5), potassium channel (PDB ID: 4UUJ), 5‐HT1B (PDB ID: 4IAQ), and human serotonin receptor (PDB ID: 5I6X) to determine anti‐nociceptive, anxiolytic, and antidepressant activities (Table 5). From these Benzaldehyde, 2‐hydroxy‐6‐methyl‐ demonstrated worthy binding affinity (Figure 6A–E) against COX‐1, COX‐2, potassium channel, 5‐HT1B, and human serotonin receptor with the highest docking score (−7.875, −5.736, −3.057, −6.35, and −6.659 kcal/mol, respectively), while all reference drugs also depicted promising binding affinity against these receptors. These docking results suggest that Benzaldehyde, 2‐hydroxy‐6‐methyl‐ may contribute to the observed pharmacological effects through modulation of inflammatory and serotonergic pathways. However, these findings are predictive and require experimental validation. Since no receptor‐binding or mechanistic assays were performed, the correlation between these *in silico* interactions and in vivo activities remains speculative and should be interpreted cautiously.

**TABLE 5:** Scores for chosen compounds from MEMPL GC–MS data using molecular docking analysis.

Compounds	Pubchem ID	2OYE	3HS5	4UUJ	4IAQ	5I6X
Diclofenac sodium			4.869			
Diazepam				−3.486	−6.095	
Fluoxetine						−8.912
3,4‐Furandiol, tetrahydro‐, cis—	641,773	−4.985	−4.308	−3.398	−4.085	−5.467
Fluoroacetic acid	5237	−4.659	−5.269	−4.313	−4.781	−4.273
2‐Furanmethanol	7361	−5.622	−4.934	−3.161	−5.053	−5.013
Ethanamine, 2‐methoxy‐N‐(2‐methoxyethyl)‐N‐methyl—	568,338	−2.958	−2.332	−1.089	−1.989	−2.135
2,4‐Dihydroxy‐2,5‐dimethyl‐3 (2H)‐furan‐3‐one	538,757	−5.743	−5.562	−5.607	−4.194	−5.607
Thymine	1135	−6.459	−5.148		−5.026	−5.082
4H‐Pyran‐4‐one, 2,3‐dihydro‐3,5‐dihydroxy‐6‐methyl—	119,838	−6.202	−5.711		−5.181	−5.456
5‐Hydroxymethylfurfural	237,332	−5.284	−5.281	−2.91	−4.096	−5.054
2‐Methoxy‐4‐vinylphenol	332	−6.284	−4.526		−5.213	−5.458
2‐hydroxy‐6‐methylbenzaldehyde	585,174	−7.875	−5.736	−3.057	−6.35	−6.659
1, 2, 3, 5‐Cyclohexanetetrol, (1.alpha., 2.beta., 3.alpha., 5.beta.)—	548,226	−6.545	−5.977		−4.831	−5.887

*Note:* 2YOE, COX‐1; 3HS5, COX‐2; 4UUJ, potassium channel; 4IAQ, 5‐HT1B; 5I6X, human serotonin receptor.

## 4 Discussion

The current study demonstrates that methanol extract of *Macaranga peltata* leaves (MEMPL) exhibits multi‐modal neuropharmacological and analgesic activities in mice, highlighting its potential (Ekor 2014) as a source of bioactive compounds for CNS and pain modulation. The diverse phytochemical profile, including flavonoids, phenols, diterpenes, and alkaloids, likely underpins its multi‐target bioactivity, though the exact constituents responsible remain to be elucidated.

The observed anxiolytic effects in the elevated plus maze and hole‐board tests may involve modulation of GABAergic neurotransmission, as flavonoids and polyphenols are known to interact with GABA_𝐴_ receptors (Bhatt et al. 2013; Herrera‐Ruiz et al. 2006). However, this mechanism remains speculative, as no direct receptor‐binding or electrophysiological evidence was obtained in the present study. Locomotor assessments demonstrated preserved exploratory activity, indicating that reduced anxiety‐like behavior was not secondary to sedation, but the possibility of subtle CNS depressant effects cannot be excluded (Kraeuter et al. 2019; Côté et al. 2018).

Similarly, the antidepressant‐like effects observed in the forced swim and tail suspension tests suggest potential involvement of serotonergic and noradrenergic pathways, consistent with prior reports on polyphenolic modulation of monoamine neurotransmission (Sarkar et al. 2021). Yet, without neurochemical or receptor‐specific assays, these remain hypothetical mechanistic interpretations. The level of antidepressant‐like and anxiolytic responses was substantially lower than the standard drugs, and therefore the activity should be considered mild to moderate rather than comparable to reference treatments.

Although diazepam and fluoxetine served as appropriate positive controls for assessing anxiolytic and antidepressant effects, the potential influence of sedation on behavioral outcomes warrants consideration. Diazepam acts via GABA_*A*_ receptor modulation and can induce sedation and reduced locomotion at higher doses. To avoid this confounding effect, a dose known to produce anxiolysis without significant motor impairment was selected. Locomotor activity tests were also conducted prior to behavioral assays to distinguish genuine anxiolytic effects from nonspecific CNS depression (Pádua‐Reis et al. 2021; Islam et al. 2025). Although MEMPL increased open‐arm exploration in the EPM and head‐dipping behavior in the HBT, these effects occurred alongside significant reductions in locomotor activity. This overlap indicates that the anxiolytic‐like response may be partially influenced by CNS depressant action rather than a selective anxiolytic mechanism.

The analgesic activity observed in acetic acid‐induced writhing and formalin‐induced paw‐licking tests points to both peripheral and central mechanisms. Inhibition of cyclooxygenase‐mediated prostaglandin synthesis may contribute to the peripheral effects, while central modulation of pain pathways is plausible (Salzer and Boehm 2019; Afsar et al. 2015). Nevertheless, these pathways were not directly measured, and the precise molecular targets remain to be experimentally verified.

Complementary virtual screening provided preliminary mechanistic insights, identifying bioactive compounds such as Benzaldehyde, 2‐hydroxy‐6‐methyl‐ with high predicted binding affinity for COX enzymes, potassium channels, and serotonin receptors. Docking results provide preliminary predictions of receptor affinity but do not establish pharmacological activity. The identification of 2‐hydroxy‐6‐methylbenzaldehyde as a possible ligand is therefore speculative. Without in vitro receptor‐binding, electrophysiology, or ex vivo functional assays, the proposed mechanisms remain unconfirmed (Alov et al. 2022).

Overall, MEMPL exhibits multi‐modal neuropharmacological and analgesic activity, but the exact molecular mechanisms remain to be established. Future studies should focus on receptor‐specific assays, neurotransmitter quantification, and structure–activity relationships to validate the hypothesized GABAergic, serotonergic, and anti‐inflammatory pathways. Such mechanistic confirmation will strengthen the translational potential of MEMPL as a source of CNS‐active and analgesic agents.

## 5 Conclusion

According to the current study, *Macaranga peltata* leaves mitigate anxiety, locomotion, and depressive‐like behavior in rodent models, while also reducing acetic acid‐ and formalin‐induced nociception. Computational studies further illustrated that 2‐hydroxy‐6‐methylbenzaldehyde binds with key neuropharmacological receptors, suggesting these effects. To fully decipher the neuroprotective potential of this promising medicinal plant, comprehensive mechanistic investigations and dose–response studies are strongly recommended.

## Author Contributions

**Shaifullah Mansur Tanzil, Ahmed Azizul Hakim, Md Tasaffiul Islam, Farhan Tanvir, Israt Jahan, Arafat Faraque, Md Amjad Hossen:** conceptualization, methodology, software, data curation, investigation, validation, formal analysis, writing – original draft. **Md. Areeful Haque, Md. Shohel Al Faruk, Kazi Ashfak Ahmed Chowdhury:** formal analysis, data curation. **Syed Mohammed Tareq, Mohammad Nazmul Islam:** conceptualization, formal analysis, project administration, resources, writing – original draft, writing – review and editing.

## Funding

The research project received no particular grants from public, commercial, or non‐profit funding organizations.

## Conflicts of Interest

The authors declare no conflicts of interest.

## Data Availability

The data that support the findings of this study are available from the corresponding author upon reasonable request.
